# Field evaluation of the photo-induced electron transfer fluorogenic primers (PET) real-time PCR for the detection of *Plasmodium falciparum* in Tanzania

**DOI:** 10.1186/1475-2875-13-31

**Published:** 2014-01-27

**Authors:** Eldin Talundzic, Mussa Maganga, Irene M Masanja, David S Peterson, Venkatachalam Udhayakumar, Naomi W Lucchi

**Affiliations:** 1 Department of Infectious Diseases, University of Georgia, Athens, GA, USA; 2 The Center for Tropical and Emerging Global Disease, University of Georgia, Athens, GA, USA; 3 Ifakara Health Institute, Dar es Salaam, Tanzania; 4 Malaria Branch, Division of Parasitic Diseases and Malaria, Center for Global Health, Centers for Disease Control and Prevention, Atlanta, GA, USA; 5 Atlanta Research and Education Foundation/VA Medical Center, Decatur, GA, USA

**Keywords:** Malaria, Molecular test, Asymptomatic malaria, Tanzania, PET-PCR

## Abstract

**Background:**

Accurate diagnosis of malaria infections remains challenging, especially in the identification of submicroscopic infections. New molecular diagnostic tools that are inexpensive, sensitive enough to detect low-level infections and suitable in laboratory settings of resource-limited countries are required for malaria control and elimination programmes. Here the diagnostic potential of a recently developed photo-induced electron transfer fluorogenic primer (PET) real-time polymerase chain reaction (PCR) called PET-PCR was investigated. This study aimed to (i) evaluate the use of this assay as a method for the detection of both *Plasmodium falciparum* and other *Plasmodium* species infections in a developing country’s diagnostic laboratory; and, (ii) determine the assay’s sensitivity and specificity compared to a nested 18S rRNA PCR.

**Methods:**

Samples used in this study were obtained from a previous study conducted in the region of Iringa, Tanzania. A total of 303 samples from eight health facilities in Tanzania were utilized for this evaluation. All samples were screened using the multiplex PET-PCR assay designed to detect *Plasmodium* genus and *P. falciparum* initially in laboratory in Tanzania and then repeated at a reference laboratory at the CDC in the USA. Microscopy data was available for all the 303 samples. A subset of the samples were tested in a blinded fashion to find the sensitivity and specificity of the PET-PCR compared to the nested 18S rRNA PCR.

**Results:**

Compared to microscopy, the PET-PCR assay was 59% more sensitive in detecting *P. falciparum* infections. The observed sensitivity and specificity were 100% (95% confidence interval (CI_0.95_) = 94-100%) and (CI_0.95_ = 96-100%), respectively, for the PET-PCR assay when compared to nested 18S rRNA PCR. When compared to 18S rRNA PCR, microscopy had a low sensitivity of 40% (CI_0.95_ = 23-61%) and specificity of 100% (CI_0.95_ = 96-100%). The PET-PCR results performed in the field laboratory in Tanzania were in 100% concordance with the results obtained at the reference laboratory in the USA.

**Conclusion:**

The PET-PCR is a new molecular diagnostic tool with similar performance characteristics as commonly used PCR methods that is less expensive, easy to use, and amiable to large scale-surveillance studies in developing country settings.

## Background

Malaria is caused by protozoan parasites of the genus *Plasmodium* that infect humans through the bite of an infected female *Anopheles* mosquito. *Plasmodium falciparum*, *Plasmodium vivax*, *Plasmodium ovale,* and *Plasmodium malariae,* all lead to malaria in humans, with the first two species causing most malaria associated mortality and morbidity. Although progress has been made towards controlling malaria worldwide, it continues to be a major public health problem [1,2]. The most recent report by the World Health Organization (WHO) estimates that 216 million cases and 655,000 deaths occurred due to malaria in 2010 [2]. The report further indicates that 106 countries are malaria endemic and up to one-half of the worldwide population is at risk of infection, with the African region accounting for 81% of malaria cases and 91% of malaria-related deaths [2].

Global malaria elimination and control programmes currently rely mostly on two diagnostic tools: immunochromatographic antigen-based rapid diagnostic tests (RDTs) and microscopy, with RDTs pioneered in the 1980s and microscopy in the late 19^th^ Century. Some of the limitations of microscopy are that evaluating stained blood smears is laborious and time consuming, it is difficult to standardize, and diagnosis of extremely low density infections is very challenging and requires very well-trained microscopists. Additionally, there is a two to three-fold discrepancy in parasite quantification between microscopists [3]. Yet microscopy is still the most widely used method, with at least 165 million smears reported to have been performed during 2010 [2], and remains the reference standard for malaria diagnosis in many malaria-endemic countries. The use of RDTs in case management and control programmes seems effective. The target antigen in over 90% of RDTs is based on detection of *P. falciparum* histidine-rich protein 2 (HRP-2) antigen. RDTs that detect lactose dehydrogenase (LDH) and aldolase are, in general, less specific in differentiating different species of human malaria parasites although recently, attempts have been made to improve the performance of these tests. The evidence that a considerable number of *P. falciparum* parasites in South America, have a natural deletion of the *HRP-2* gene [4] raises concerns about the use of RDTs in some settings. Furthermore, new data show that caution needs to be taken with the positive predictive values of RDTs [5] and that many tests falls below the desired 95% sensitivity/specificity target in poor environmental conditions and in children under the age of five years [5]. Nonetheless, RDTs are still a practical usable diagnostic method in the management of febrile illness in remote regions [6,7].

There is an urgent need for developing robust field usable molecular tests with high level of sensitivity and specificity for use in large-scale screening of samples from malaria surveillance studies especially those that are focused on malaria control and elimination. This need has become particularly apparent in malaria containment projects that are focused on eliminating malaria in regions where parasites resistant to artemisinin-based combination therapy (ACT) have evolved [8-10]. Advancements in molecular technologies provide a continually evolving and relatively low cost system for high-throughput screening of malaria in surveillance studies [11-13]. Molecular methods are more reliable than traditional microscopy and RDTs in accurately diagnosing the species of malaria parasites and detecting low parasitaemia levels. When considering the development of tools for large-scale field application, it is desirable to consider cost, robustness and ease of use. Recently, a novel real-time PCR assay that utilizes self-quenching primers for the detection of *Plasmodium spp.,* and *P. falciparum* was developed [13]. Therefore, this PCR assay does not require internal dual-labelled probes, which are usually expensive, or use intercalating dyes, which are often non-specific. The PET-PCR is an attractive molecular assay for malaria detection, especially in surveillance studies in endemic countries where this methodology can be more readily adopted than other probe-based real-time PCR methods. The study herein aimed to evaluate the PET-PCR assay for the detection of malaria parasites in a field laboratory in Tanzania, and compare its performance to microscopy and a widely used nested 18S rRNA PCR.

## Methods

### Study area and samples

**Figure 1:**
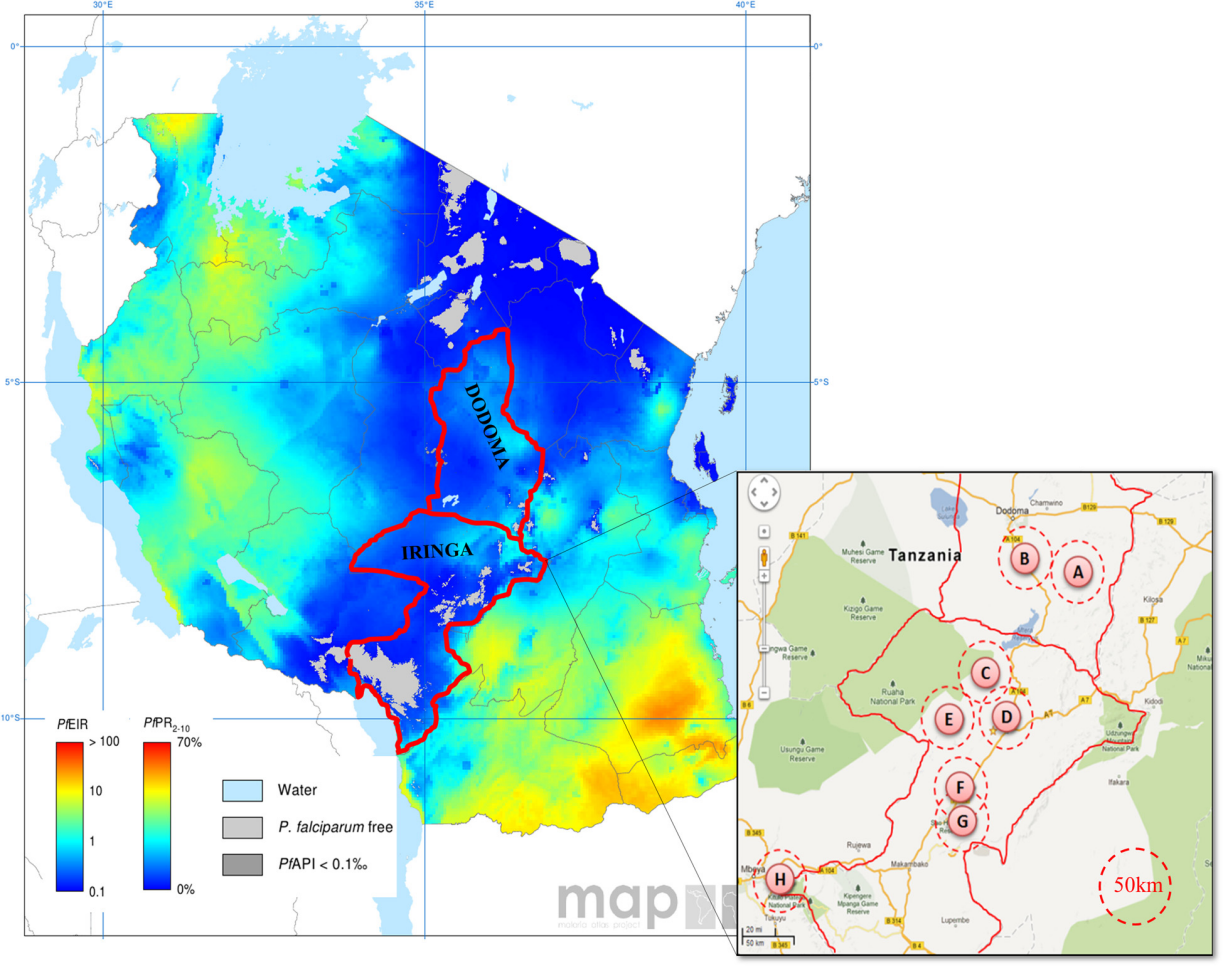
**Regions in Tanzania screened for species-specific malaria infection using PET-PCR.** Shown is the 2010 map of spatial distribution of *Plasmodium falciparum* malaria endemicity and entomological inoculation rate (EIR) [14] in the region of Iringa where samples used in this study were obtained. Samples were collected from eight health facilities as shown (A-H). *Plasmodium falciparum* prevalence (PfPR) and entomological inoculation rate (PfEIR) for selected region are shown, 0% > PfPR ≤5% and 0.1 > PfEIR ≤1, respectively.

Samples used in this study were obtained from a previous study conducted in the Iringa region of Tanzania during the years of 2010–2011 for assessing the accuracy of RDTs. These were collected from the following eight health facilities: (A) Tosamaganga, (B) Mlowa, (C) Kimande, (D) Usokami, (E) Idodi, (F) Mafinga, (G) Kibao, and (H) Igomaa. The region and districts are shown in Figure 1 on the 2010 map of spatial distribution of *P. falciparum* malaria endemicity and entomological inoculation rate (EIR) [14]. The two districts from which the samples were collected have a *P. falciparum* prevalence (PfPR) and entomological inoculation rate (PfEIR) of ≤5% and ≤1, respectively. A blood smear and dried blood spot (DBS) samples were collected as part of the original study. A subset of 303 DBS samples, randomly selected from the eight health facilities, was made available for use in this study.

### Ethics statement

The parent study was reviewed by IHI and granted a national ethical permit from the Tanzanian National Institute for Medical Research (NIMR). It was determined by CDC IRB to be a non-research activity with no requirement for consent from participants.

### Microscopy

Both thick and thin smears were prepared per sample. Depending on the health facility, the blood smears were stained using 10% or 5% Giemsa solution buffered to pH 7.2, and stained using a standard protocol. A blood smear was considered negative when no parasite was detectable after examining 100 high power fields. For positive smears, parasites were counted in reference to 200 white blood cells. Blood smears were first read by a district laboratory technician in charge of laboratory services in the rural districts where samples were collected. The slides were then sent to a reference microscopy laboratory at IHI in Bagamoyo, Tanzania. Any discordant reading between the district and reference microscopy reading was resolved by a third reading by a senior technician from the Muhimbili University of Health and Allied Sciences (MUHAS).

### DNA extraction

DNA was isolated from DBS samples at IHI using the commercially available QIAamp DNA Mini Kit (QIAGEN, Valencia, CA, USA). Genomic DNA was eluted with 100 μl of elution buffer and stored at −20°C for use in PCR assays.

### PET-PCR Assay

The PET-PCR assay was performed as previously described with a few modifications [15]. Briefly, amplification of the species (*P. falciparum)* and genus (*Plasmodium)* DNA targets was performed in a 20 μl reaction containing 2X TaqMan Environmental Master Mix 2.0 (Applied BioSystems), 125 nM of forward and reverse primers, except the *P. falciparum* labelled primer whose concentration was 62.5 nM, and 2 μl of DNA template. The reactions were performed under the following cycling parameters: initial hot-start at 95°C for 10 min, followed by then 45 cycles of denaturation at 95°C for 10 sec, annealing at 60°C for 40 sec. The correct fluorescence channel was selected for each fluorescently labelled primer set and the cycle threshold (CT) values recorded at the end of the annealing step. A cut-off CT value of 40 was used to indicate a positive result. All samples were tested in duplicates and repeated when necessary. The DNA stocks that were used in Tanzania were also utilized in the USA reference laboratory. The Stratagene Mx3000P real-time PCR system was used to test all samples in both Tanzania and the USA.

### Nested 18S rRNA PCR

Nested 18S rRNA PCR tests have been shown to be superior in their sensitivity and specificity over microscopy and are commonly used as reference tests for molecular assays. Here the nested 18S rRNA PCR assay described by Singh *et al.* was used as the reference standard [16]. Briefly, reactions were performed using 1 uL DNA template in 20 μl total volume containing 1X buffer, 2.5 mM MgCl_2_, 200 μM dNTPs, 200 nM primers, and 1.25 units of Taq Polymerase (New England Biolabs, Ipswich, MA, USA). The products were analysed for the appropriate size on a 2% agarose gel. All nested 18S rRNA PCR was performed at the Center for Disease Control and Prevention (CDC) laboratory.

### Data analysis

Data were analysed using the R software for statistical computing [17]. For calculating sensitivity and specificity, including the positive predictive value(s) (*ppv)* and negative predictive value(s) (*npv)*:

Sensitivity = # of true positives / (# of true positives + # of false negatives)

Specificity = # of true negatives / (# of true negatives + # of false positives)

Positive predictive value (ppv) = # of true positives/(# of true positives + # of false positives)

Negative predictive value (npv) = # of true positives/(# of true negatives + # of false negatives)

## Results

### Assessment of PET-PCR performance at a local laboratory in Tanzania

Twenty-seven samples out of the 303 tested were found to be positive for both *P. falciparum* and *Plasmodium* spp. by PET-PCR when performed at the IHI laboratory in Tanzania. The same samples were retested at the CDC laboratory and 100% agreement was found between the PET-PCR results done at IHI and CDC.

### Comparison of PET-PCR and microscopy

Microscopy detected 11 positive samples (3.63%) among the 303 samples tested. These microscopy positive samples were confirmed to be positive by PET-PCR. In addition, 16 microscopy-negative samples were found to be positive for both *P. falciparum* and *Plasmodium* spp. by PET-PCR (Table 1).

**Table 1:** Number of positive samples by health facilities as detected by PET-PCR, nested 18S rRNA, and microscopy

Health facilities*	**A**	**B**	**C**	**D**	**E**	**F**	**G**	**H**	**Total**
**Total no of samples**	**51**	**33**	**36**	**31**	**46**	**23**	**59**	**24**	**303**
PET-PCR positive	7	7	4	0	7	0	1	1	27
Nested 18S rRNA	7	7	4	0	7	0	1	1	27
Microscopy positive	1	3	4	0	3	0	0	0	11

*Health facilities: (A) Tosamaganga, (B) Mlowa, (C) Kimande, (D) Usokami, (E) Idodi, (F) Mafinga, (G) Kibao, and (H) Igomaa. PET-PCR results shown are for both *Plasmodium falciparum* and *Plasmodium* species*.*

### Sensitivity and specificity of PET-PCR and microscopy

To determine the sensitivity and specificity of the multiplex PET-PCR assay and microscopy, all 27 positive samples and a subset of 117 PET-PCR and microscopy negative samples were tested using a nested 18S rRNA PCR method as a reference test [16]. The samples were randomly and in a blinded fashion selected with an average of 14 samples from each health facility. The data showed 100% sensitivity (CI_0.95_ = 94-100%) and specificity (CI_0.95_ = 96-100%) for the PET-PCR assay and 40% sensitivity (CI_0.95_ = 23-61%) and 100% specificity (CI_0.95_ = 96-100%) for microscopy (Table 2).

**Table 2:** Sensitivity and specificity of the PET-PCR assay and microscopy when compared to nested 18S rRNA PCR

	**Reference standard**		
**Test result**	Nested 18S rRNA PCR		
Multiplex PET PCR	Present (+)	Absent (−)	
Test positive (+)	27	0	27 (18.75%)
Test negative (−)	0	117	117 (81.25%)
	27 (18.75%)	117 (81.25%)	144 (100.00%)
		**95% confidence interval:**	
		**Lower limit**	**Upper limit**
**Sensitivity**	100.00%	94.04%	100.00%
**Specificity**	100.00%	96.10%	100.00%
*PPV*	100.00%	100.00%	100.00%
*NPV*	100.00%	100.00%	100.00%
	**Reference standard:**		
**Test result:**	Nested 18S rRNA PCR		
Microscopy	Present (+)	Absent (−)	
Test positive (+)	11	0	11 (7.63%)
Test negative (−)	16	117	133 (92.36%)
	27 (18.75%)	117 (81.25%)	144 (100.00%)
		**95% confidence interval:**	
		**Lower limit**	**Upper limit**
**Sensitivity**	40.70%	23.00%	61.00%
**Specificity**	100.00%	96.00%	100.00%
*PPV*	100.00%	67.90%	100.00%
*NPV*	87.70%	80.90%	92.80%

## Discussion

Of the 303 samples from the eight health facilities, 8.9% were found to be positive for *P. falciparum* by both PET-PCR and nested 18S rRNA PCR. In contrast, only 3.6% were positive by microscopy revealing a clear underestimation of malaria infection when diagnosed by microscopy. The PET-PCR method detected a large number of submicroscopic infections. This is in agreement with previous studies that reported the detection of a greater number of submicroscopic infections by molecular methods [18-21]. Indeed, the PET-PCR assay compared favourably to the molecular reference standard, nested 18S rRNA PCR, with 100% agreement between both methods. However, PET-PCR offers important advantages over standard nested PCR, such as affordability, faster turnaround time, less chance for DNA cross contamination due to lack of post PCR manipulations, and is overall better suited for large scale screening. In addition, the assay was shown to be comparable to the TaqMan-based real-time PCR method described by Rougemont et al. [22] in which both the PET-PCR and Rougemont assays showed a detection limit as low as 3.2 parasites/·L [15]. This detection limit is also comparable to the reference standard nested 18S rRNA PCR where the detection limit ranged between 0.64-3.2 parasites/·L [15]. Due to the self-quenching nature of the PET primers this assay does not require expensive probes, making it a relatively less expensive method. The estimated average PET-PCR reagent cost for this study was around US $2 per reaction. In addition, the reconstituted PET primers and master mix can be stored together at 4°C for up to a month or if the primers are kept lyophilized can be stored until the end-user is ready to use them. These practical advantages, along with the relatively low cost, make PET-PCR a sustainable new method for malaria detection for large scale screening of field samples. The multiplex PET-PCR assay was first tested in a local diagnostic laboratory in Bagamoyo, Tanzania and then repeated at the CDC laboratory. A complete agreement of results was obtained between the two settings in performing the PET-PCR assay, indicating that this assay can be successfully utilized in a field laboratory in malaria-endemic countries. In addition, most standard real-time PCR machines will be appropriate to carry out the assay.

Microscopy and RDTs still remain the most appropriate methods for primary diagnosis of malaria in many malaria-endemic countries. However, as the goal moves towards malaria elimination, newer tools that are able to detect all malaria cases, including low density infections not detectable by microscopy or RDTs, and that are applicable to large-scale screening, are required. The PET-PCR assay described herein provides such a tool as it has performance characteristics similar to other commonly used molecular methods and can be used in a developing country setting. While this assay cannot yet be used in most primary health facilities in many malaria-endemic regions, many reference and research laboratories are already equipped for molecular testing such as PET-PCR.

## Conclusion

The PET-PCR assay provides a relatively rapid and affordable new molecular method for large-scale screening of surveillance samples in support of malaria control and elimination programmes in malaria-endemic countries.

## Competing interests

The authors have declared that they have no competing interests.

## Authors’ contributions

ET, MM and NWL carried out the molecular assays. ET, NWL and VU drafted the manuscript. IM provided the samples for the study and helped draft the manuscript. ET, NWL, IM, DP, and VU conceived and designed the study. All authors have read and approved the final manuscript.
